# Dizziness and health-related quality of life among older adults in an urban population: a cross-sectional study

**DOI:** 10.1186/s12955-021-01864-z

**Published:** 2021-10-02

**Authors:** Ellen Lindell, Lena Kollén, Mia Johansson, Therese Karlsson, Lina Rydén, Madeleine Mellqvist Fässberg, Hanna Falk Erhag, Ingmar Skoog, Caterina Finizia

**Affiliations:** 1 Department of Otorhinolaryngology, Head and Neck Surgery, Institute of Clinical Sciences, Sahlgrenska Academy, Sahlgrenska Universitetssjukhuset, Gothenburg University, Gröna Stråket 5, 413 45 Gothenburg, Sweden; 2 Department of Otorhinolaryngology, Region Västra Götaland, Sahlgrenska University Hospital, 413 45 Gothenburg, Sweden; 3 Department of Occupational Therapy and Physiotherapy, Institute of Clinical Sciences, Sahlgrenska Academy, Gothenburg University, 413 45 Gothenburg, Sweden; 4 Department of Oncology, Institute of Clinical Sciences, Sahlgrenska Academy, Gothenburg University, 413 45 Gothenburg, Sweden; 5 Neuropsychiatric Epidemiology Unit, Institute of Neuroscience and Physiology Sahlgrenska Academy, Centre for Ageing and Health (AgeCap), University of Gothenburg, Gothenburg, Sweden

**Keywords:** Dizziness, Aged, Health-related quality of life, Sense of coherence, Self-rated health, Quality of Life

## Abstract

**Background:**

Dizziness is a common complaint among older adults and may affect quality of life in a negative way. The aim of this study was to assess health-related quality of life (HRQL), sense of coherence (SOC), self-rated health (SRH) and comorbidity in relation to dizziness, among older persons from an urban population.

**Methods:**

The study is part of the Gothenburg H70 Birth Cohort Studies (H70). A cross-sectional population-based sample including 662 79-years-olds (404 women, 258 men, 62% response rate) were surveyed with questions regarding dizziness, imbalance, comorbidities and general health. HRQL was assessed using the 36-item Short Form-36 Health Survey (SF-36) and SOC with the 13-items questionnaire Sense of Coherence (SOC-13**).**

**Results:**

Half of the participants reported problems with dizziness (54%). Dizziness was negatively associated with HRQL, including after adjusting for comorbidities, especially in the physical domains of SF-36. Having dizziness was also associated with poorer SRH, tiredness and comorbidity among both men and women. SOC (mean total score), however, did not differ between dizzy and non-dizzy participants.

**Conclusions:**

Dizziness was negatively associated with HRQL, also after adjusting for comorbidities. Identification and treatment of dizziness, when possible, are important because reduction of dizziness symptoms may potentially help to enhance overall well-being in this age group.

## Background

Dizziness is a common complaint among older individuals. Approximately one third have impairment of balance or dizziness at age 75 and reports of dizziness increase with advanced age [1, 2]. Dizziness among older adults may have multiple etiologies, is often associated with comorbidity and frailty, and is sometimes viewed as part of the geriatric syndrome and aging [3–5].

Worldwide, life expectancy has grown remarkably during the last two decades and is presently 84 years in women and 80 years in men in Sweden [6]. By 2060, approximately one-fourth of the Swedish population is estimated to be over 65 years [6]. With an aging population, prevalence of dizziness and unsteadiness is likely to become more frequent in the forthcoming years. Reasons for having dizziness in higher ages can be many where benign peripheral vestibular disorder and decreased postural control can be affected and impact balance or induce dizziness. With higher age, factors affecting balance such as multimorbidity, neuropathy, stroke, cardio vascular disease and impairment of joints like back pain become more common. Having dizziness can be a frightening condition and may increase risk of falls as well as enhance fear of falling and affect quality of life [7, 8]. Furthermore, with higher life expectancy and longer lives, health-related quality of life (HRQL) may become a key outcome in surveys investigating older people.

HRQL is a multidimensional construct and used as an indicator of broad physical and mental health. It specifically refers to the physical, psychological and social domains of health, seen as distinct areas that are influenced by a person’s experiences, beliefs, expectations and perceptions [9, 10]. HRQL can be used to monitor changes of a disease as well as to assess the impact of illness burden of clinical conditions within general populations [11, 12].

The ability to stand and move safely is essential for living independently. Having dizziness, as well as impaired balance, influences functional abilities such as moving around or walking and the inability to move safely may induce fear of losing balance and falling [13]. Having dizziness has been shown to correlate with increased number of comorbidities and reduced HRQL [3, 5, 14–18]. Enhanced fitness level and being physically active, on the other hand, are associated with higher HRQL among older people [14, 19], meanwhile those with dizziness have been found to be less physically active and to have more diseases [3, 20]. Whether the reduction in HRQL is due to comorbidities, the dizziness symptom, or less physical performance is unclear. The Short-form 36 Health Survey (SF-36) is one of the most used instruments to assess HRQL worldwide and has been proven useful in monitoring health, estimating burdens of diseases, and evaluating medical treatment effects [21].

Self-rated health (SRH) is also a widely used, albeit more poorly understood, measurement when evaluating health. SRH is based on asking individuals to evaluate their global health status on a four- or five-point scale, although the exact wordings and response options may vary [22, 23]. There is widespread agreement that this simple global question provides a useful summary of how older people perceive their overall health status, and in some cases, SRH has turned out to be a better predictor of mortality than objective health indicators [24, 25]. SRH can be seen as a sensitive, but non-specific way, to assess health and predict outcomes [26].

Antonovsky developed the theory of sense of coherence (SOC) in the 1970s focusing on factors that support human health and well-being. SOC refers to the ability to utilize internal and external resources to cope with stressors and maintain health. The concept aims to explain how people remain healthy rather than become ill. Antonovsky introduced three components: *Comprehensibility*, the extent to which one perceives events as making sense; *Manageability*, the extent to which one feels he or she can cope; and *Meaningfulness*, the extent to which one feels that life makes sense and that challenges are worthy of commitment. A high SOC has been shown to promote health and even reduce mortality and is related to good health especially in the mental dimension [27, 28].

Only a few studies have focused on dizziness in relation to HRQL and SOC [29, 30], where investigations are carried out on patients seeking medical care in balance clinics. A Swedish survey of patients suffering from vestibular disorders showed an association between unsteadiness, fall tendency and lower scores on SOC [29]. None of the studies on SOC and dizziness have been population-based, targeting older people with general dizziness/unsteadiness.

The aim of this study was to assess HRQL, SOC, SRH and comorbidity in relation to dizziness among older men and women in an urban population.

## Methods

### Study population

This study was part of the fifth cohort of the geriatric population-based Gothenburg H70 Birth Cohort Studies, that studies health-related factors in older adults. Persons were selected from an urban population born in 1930 through systematic sampling based on date of birth using the Swedish National Population Register. Invited people included those living in either ordinary or special housing. A total of 1063 individuals (659 women, 404 men), all aged 79 years old, were invited to participate and 662 individuals (404 women and 258 men; 62% response rate) agreed to participate in the multidisciplinary survey. The study was conducted in 2009–2010. The participants were interviewed by a research nurse or physician at the neuropsychiatric outpatient clinic at Sahlgrenska University Hospital in Gothenburg, Sweden, or in the participants’ home.

### Dizziness

Study-specific questions concerned dizziness, general health and tiredness. The main variable was the question concerning dizziness: “Do you have any problems with dizziness, impaired balance or unsteadiness?”. The response alternatives were yes/no. If “yes”, further questions included; “Duration of dizziness?” (seconds/minutes/hours); “For how long have you had dizziness?” (< 6 months, 6 months–2 years, > 2 years). “Do you have such dizziness that it hinders you in your daily activities? (yes/no). Dizziness triggered by; “Rising from supine to sitting position?” (yes/no), “When lying on the side?” (yes/no). “When turning the head?” (yes/no), “When you exercise? (yes/no), “By positional changes?” (yes/no). A total of 647 people (395 women, 252 men) answered the questions regarding dizziness and were included in this study. All questions and questionnaire were in Swedish language.

### Characteristics of the participants

The participants were asked about their current marital status, type of living, if they were dependent on help in activities of daily living (ADL) and instrumental activities of daily living (IADL) according to ten different functions from the Katz scale [31].

### HRQL

HRQL was assessed with SF-36 which consists of 36 questions distributed over eight subscales: physical function (PF), role limitation due to physical problems (RP), bodily pain (BP), general health (GH), vitality (VT), social function (SF), role limitation due to emotional problems (RE) and mental health (MH) [21]. Every component is standardized and the scores range from 0 (worst) to 100 (best). Low scores represent low HRQL. The SF-36 is a widely used instrument for measuring self-perceived physical and mental health status. Validity and reliability have been evaluated and confirmed in prior studies [32]. The instrument is translated to Swedish and has been validated in a representative sample of the population [33]. In this study a total of 471 participants (284 women, 187 men) answered the SF-36 questionnaire and mean and standard deviation (SD) was calculated for every domain of the SF-36.

### SOC

The SOC instrument was developed by Antonovsky to measure overall coping capacity during stressful situations and circumstances in life [34]. The 13 items questionnaire (SOC-13) used in this study is a shorter version of the original 29-item instrument. It is used to study three domains, namely; comprehensibility, manageability and meaningfulness. The total score ranges from 13 to 91 where each question is scored on a 7-point scale. The higher the score, the stronger the SOC. The validity of the questionnaire is reported to be acceptable and test–retest stable [35]. In this study a total of 452 participants (272 women, 180 men) answered the SOC-13.

### SRH, tiredness and comorbidity

SRH was measured using the question, “How is your general health?” with response options ranging from “very good”, “good”, “poor” to “very poor”. In addition, respondents also answered whether they felt well or generally tired (yes/no). Regarding diseases, the participants were asked about current conditions and diseases: “Have you been told by a medical doctor or nurse that you have or have had, any of the following diseases or disorders?” and were asked according to a list of conditions including: diabetes, hypertension, hyper or hypothyroidism, lung disease (chronic bronchitis, asthma, chronic obstructive pulmonary disease), cardiac infarction/heart failure, prior stroke, vision and hearing impairment and joint/back problems.

### Statistical methods

Mean, median and 95% confidence intervals (CI) or standard deviation (SD) of the mean were used for descriptive purposes. Mean and CI or SD were used for continuous variables while number and percent were used for categorical variables. Tests of differences between the group with and without dizziness, and between men and women included Fisher’s exact test and logistic regression for dichotomous variables, Mantel–Haenszel chi square test for ordered variables, Chi square test for non-ordered variables and *t* tests for continuous variables. Significance was always reported for two-tailed tests and the significance level used was 5%.

Backward multivariable linear regression was conducted (*p* < 0.01) to identify factors and conditions influencing HRQL (SF-36 domains, 8 domains) as dependent variables. Beta-estimate, CI, *p *value and R-square were presented. The software used for the statistical analysis was SPSS 24 or a statistics program package developed at the Department of Geriatrics at University of Gothenburg (GIDSS for Windows).

### Ethical considerations

The study was conducted according to the Declaration of Helsinki and was approved by the Regional Ethical Review Board in Gothenburg, Sweden. All participants gave their informed written consent before inclusion in the study.

## Results

### Dizziness and unsteadiness

More than half of the sample of 79-year-olds reported problems with dizziness or unsteadiness and no significant differences in prevalence were observed between men and women (n = 125, 50% of the men; n = 222, 56% of the women; *p* = 0.12). The majority of the participants with dizziness reported problems with dizziness for more than six months (82% of the women, 78% of the men) and more than half of the participants with dizziness reported problems with dizziness daily or weekly (56% of the women, 57% of the men). Dizziness lasting for only seconds was the most common duration (n = 54, 65% of the men; n = 90, 65% of the women) whereas dizziness lasting minutes (n = 20, 14% of the women; n = 16, 20% of the men) or hours (n = 28, 20% of the women; n = 12, 15% of the men) where not so common, also reported elsewhere [18]. Dizziness triggered by rising up from supine position was the most common symptom (n = 46, 61% of the men; n = 68, 50% of the women) followed by dizziness triggered by positional changes (n = 24, 34% of the men; n = 48, 38% of the women) and by turning the head (n = 16, 24% of the men; n = 26, 22% of the women). More than one quarter of the participants with dizziness (n = 22, 23% of the men, n = 48, 31% of the women) reported that dizziness had an impact on their daily activities and about one fifth (n = 13, 19% of the men; n = 24, 20% of the women) reported dizziness by physical exercise. Dizziness when lying on the side was reported by 8 women (7%) and 2 (3%) men.

### Characteristics

There was no significant difference in type of housing or marital status between dizzy and non-dizzy individuals (Table 1). Persons with dizziness had higher levels of IADL dependency than persons without dizziness (Table 1). Some sex differences were seen between the distribution of diseases where a greater percentage of men had vascular diseases (stroke, heart disease) and a greater percentage of women reported thyroid disease and joint/back pain (Table 2).

**Table 1:** Characteristics of study population

	Women (n = 395)		Men (n = 252)		Women and men (n = 647)	
	No dizziness n = 173	Dizziness n = 222	No dizziness n = 127	Dizziness n = 125	No dizziness n = 300	Dizziness n = 347
*Marital status, n (%)*						
Single	18 (11)	12 (6)	14 (11)	13 (10)	32 (11)	25 (7)
Married	57 (33)	74 (34)	89 (70)	82 (65)	146 (49)	156 (46)
Divorced	23 (13)	35 (16)	7 (6)	10 (8)	30 (10)	45 (13)
Widow	73 (43)	95 (44)	17 (13)	20 (16)	90 (30)	115 (34)
*Type of housing, n (%)*						
Apartment	115 (67)	145 (56)	70 (56)	74 (60)	185 (63)	219 (65)
Private house	51 (30)	60 (28)	53 (42)	47 (38)	104 (35)	107 (32)
Sheltered living	5 (3)	9 (4)	2 (2)	3 (2)	7 (2)	12 (4)
*Dependent in help with, n (%)*						
Cleaning the house	28 (16)	61 (29)**	22 (17)	29 (23)**	50 (17)	90 (26)**
Shopping	24 (14)	37 (17)	8 (6)	22 (18)**	32 (11)	59 (17) *
Transport	23 (13)	40 (18)	6 (5)	17 (14)*	29 (10)	57 (17)*
Cooking	12 (7)	16 (7)	7 (6)	18 (15)*	19 (6)	34 (10)
Bathing	12 (7)	18 (8)	3 (2)	12 (10)*	15 (5)	30 (9)
Dressing	11 (6)	6 (3)	2 (2)	5 (4)	13 (4)	11 (3)
Toileting	6 (3)	3 (1)	2 (2)	3 (2)	8 (3)	6 (2)
Transferring	4 (2)	4 (2)	2 (2)	1 (1)	6 (2)	5 (1)
Continence	7 (4)	8 (4)	3 (2)	3 (2)	10 (3)	11 (3)
Eating	2 (1)	2 (1)	1 (1)	0	3 (1)	2 (1)
*Number of ADL*^a^*dependency*						
0	141 (82)	151 (69)	104 (83)	92 (74)	245 (82)	243 (71)
1	10 (6)	34 (16)	13 (10)	7 (35)	23 (8)	41 (12)
2–3	9 (5)	20 (9)	6 (5)	16 (73)	15 (5)	36 (10)
4–8	12 (7)	14 (6)	3 (2)	9 (75)	15 (5)	23 (7)
		ns		*p* < 0.05		*p* < 0.01

^a^*ADL* activities of daily living. *p *values based on and Fisher’s exact test (dependence of help for ADL and instrumental ADL), Chi square test (marital status, type of housing) and Mantel–Haenszel chi square test (number of ADL dependencies), comparing dizzy and non-dizzy individuals, *p < 0.05, **p < 0.01, ***p < 0.001

**Table 2:** Frequency of conditions and diseases among men and women in the study cohort

	Women (n = 395) Frequency, n (%)	Men (n = 252) Frequency, n (%)	*p* value
Diabetes	49 (12)	41 (16)	0.2
Hypertension	228 (57)	152 (59)	0.6
Thyroid disease	62 (15)	15 (6)	0.001
Lung disease	51 (13)	31 (12)	0.8
Heart disease	89 (22)	82 (32)	0.008
Stroke/TIA^a^	65 (16)	62 (24)	0.015
Vision impairment	114 (28)	73 (28)	1.0
Hearing impairment	120 (30)	86 (33)	0.4
Joint/back problems	198 (49)	102 (40)	0.02

^a^*TIA* transient ischemic attack. *p* values comparing differences between men and women

### HRQL

Being dizzy was negatively associated with HRQL. The negative association between dizziness and HRQL was seen for every domain except RE among women and MH among men, with the largest impact in the physical domains (Table 3). After adjustment for diseases and conditions, being dizzy still had significant impact on HRQL measured with SF-36 in five domains (PF, BP, GH, VT, and SF) (Table 4). Joint/back problems and vision were the factors influencing HRQL the most in the regression analyses (Table 5). Men had slightly higher scores compared to women on all SF-36 subscales, but significant sex differences were only seen in the PF (*p* < 0.01), BP (*p* < 0.01), and MH (*p* < 0.05) domains (Table 6).

**Table 3:** HRQL (SF-36), SOC (SOC-13), SRH and tiredness among women and men with and without dizziness

	Women		Men		Women and men	
	No dizziness	Dizziness	No dizziness	Dizziness	No dizziness	Dizziness
*SF-36, mean (SD)*						
n=	132	152	94	93	226	245
Physical functioning						
Role physical	69.7 (24.6)	57.2 (24.0) ***	77.6 (20.0)	65.6 (24.0) **	73.0 (23)	60.3 (24.9) ***
Bodily pain	65.5 (41.6)	52.2 (44.0) **	73.8 (37.6)	55.6 (42.1) **	69.0 (40.0)	53.5 (43.3) ***
General health	70.9 (27.3)	53.0 (24.1) ***	76.7 (25.3)	65.0 (27.3) **	73.3 (26.7)	57.5 (25.9) ***
Vitality	70.0 (18.7)	56.5 (20.6) ***	68.1 (18.7)	60.6 (21.5) *	69.2 (18.7)	58.0 (20.0) ***
Social functioning	68.1 (21.6)	55.1 (24.4) ***	70.6 (20.6)	59.7 (23.1) **	69.2 (21.2)	56.8 (23.9) ***
Role emotional	86.3 (20.1)	76.3 (25.7) **	87.8 (20.3)	81.1 (25.0) *	86.9 (20.6)	78.1 (25.5) ***
Mental health	76.1 (39.1)	67.6 (40.5)	79.6 (33.0)	68.4 (39.7) *	77.6 (36.7)	67.9 (40.1) **
	80.9 (18.3)	73.0 (21.8) **	84.5 (17.5)	80.7 (16.0)	82.4 (18.0)	75.9 (20) **
*SOC-13, mean (SD)*						
n=	126	146	98	82	228	224
SOC-13 total	75.4 (11.1)	74.2 (10.3)	75.7 (8.37)	74.7 (9.31)	75.5 (9.99)	74.4 (9.97)
SOC:						
Comprehensibility	28.0 (4.60)	28.0 (4.26)	28.7 (3.59)	28.0 (3.95)	28.3 (4.19)	28.0 (4.15)
Manageability	23.0 (3.96)	22.6 (3.51)	23.0 (3.07)	22.9 (3.01)	23.0 (3.59)	22.7 (3.34)
Meaningfulness	24.2 (4.61)	24.0 (4.20)	24.6 (3.09)	23.8 (4.19)	24.4 (4.02)	23.8 (4.19)
*SRH and tiredness, n (%)*						
Very good	54 (32)	22 (10)	39 (31)	12 (10)	93 (31)	34 (10)
Good	103 (61)	154 (70)	79 (63)	95 (77)	182 (62)	249 (72)
Pretty poor	12 (7)	39 (18)	6 (5)	14 (11)	18 (6)	53 (16)
Very poor	1 (1)	5 (2)	2 (2)	3 (2)	3 (1)	8 (2)
		*p* < 0.0001		*p* < 0.001		*p* < 0.0001
Not feeling well	22 (13)	62 (29) **	15 (12)	31 (25) **	37 (13)	93 (27) ***
Feeling tired	61 (36)	113 (52) **	35 (28)	57 (46) **	96 (32)	170 (50) ***

Health-related quality of life (HRQL) measured with Short form Health Survey questionnaire (SF-36), Sense of coherence (SOC-13), self-rated health (SRH) and tiredness among women and men with and without dizziness

*p* values based on *t* test (SF-36, SOC-13), Mantel–Haenszel chi square test (SRH) and Fisher’s exact test (feeling tired, not feeling well), comparing dizzy and non-dizzy individuals (SF-36, SOC-13), *p < 0.05, **p < 0.01, ***p < 0.001.

The maximum score for SF-36 is 100 for every domain, higher scores indicate better HRQL. The maximum total score for SOC-13 is 91 (range 13–91), higher scores indicate higher sense of coherence

**Table 4:** Difference in mean in the domains of the Short form Health Survey questionnaire (SF-36)

	Physical functioning	Role physical	Bodily pain	General health	Vitality	Social functioning	Role emotional	Mental health
*Dizziness, unadjusted*								
Mean	− 12.4	− 15.1	− 15.5	− 11	− 12.3	− 8.5	− 9.1	− 6.3
CI^a^	− 8.1, − 16.8	− 7.5, − 22.7	− 10.7, − 20.3	− 7.3, − 14.6	− 8.2, − 16.4	− 4.3, − 12.7	− 2.0, − 12.7	− 2.8, − 9.7
*p *value	< 0.0001	0.0001	< 0.0001	< 0.0001	< 0.0001	0.0001	0.0001	0.0004
Dizziness, adjusted*								
Mean	− 6	− 7.4	− 8.2	− 4.2	− 6.4	− 4.9	− 5.4	− 2.7
CI	− 10.3, − 1.6	− 15.5, − 0.7	− 12.6, − 3.6	− 7.6, − 0.5	− 10.4, − 2.4	− 9.3, − 0.5	− 13, − 2.1	− 6.0, − 0.7
*p* value	0.007	0.07	0.0005	0.02	0.002	0.03	0.2	0.1

Difference in mean in the domains of the Short form Health Survey questionnaire (SF-36) measuring health-related quality of life between dizzy and no dizzy participants, unadjusted and adjusted* values

^a^*CI* confidence interval

*Adjusted for sex, sense of coherence (SOC)-13 total score, diabetes, hypertension, hypothyroidism, lung disease (chronic obstructive pulmonary disease, asthma, chronic bronchitis), heart disease, stroke/transient ischemic attack, vision impairment, hearing impairment, joint problems and sum of seven diseases in a linear regression

**Table 5:** Multivariable linear regression between different domains of SF-36 and SOC-13, gender, conditions and diseases

SF-36 domain	Variable	Estimate	CI	R-square	*p* value
Physical functioning	Intercept	88.3			
	Female sex	− 5.9	− 10.2, − 1.6	0.24	0.007
	Dizziness	− 6	− 10.3, − 1.6		0.007
	Diabetes	− 10.7	− 16.9, − 4.4		0.0009
	Lung disease	− 13.4	− 19.7, − 7.1		< 0.0001
	Vision impairment	− 6.7	− 11.1, − 2.3		0.003
	Joint/back problem	− 14.8	− 19.5, − 10.2		< 0.0001
Role physical	Intercept	30.3			
	SOC− 13 total score	0.7	0.26, 1.1	0.14	0.001
	Heart disease	− 12.8	− 21.8, − 4.0		0.005
	Vision impairment	− 10.7	− 18.8, − 2.6		0.01
	Joint/back problem	− 16.9	− 25.1, − 8.7		< 0.0001
Bodily pain	Intercept	56.7			
	Dizziness	− 8.2	− 12.8, − 3.6	0.31	0.0005
	SOC− 13 total score	0.4	0.18, 0.63		0.0004
	Vision impairment	− 6.3	− 11.0, − 1.7		0.008
	Joint/back problem	− 23	− 28.5, − 18.7		< 0.0001
General health	Intercept	60.9			
	SOC-13 total score	0.3	0.1, 0.5	0.36	0.0003
	Diabetes	− 6.9	− 11.8, − 1.9		0.007
	Lung disease	− 7.05	− 12.1, − 2.0		0.006
	Heart disease	− 10.5	− 14.4, − 6.7		< 0.0001
	Vision impairment	− 5.6	− 9.0, − 10.1		0.002
	Joint/back problem	− 13.6	− 17.1, − 10.1		< 0.0001
Vitality	Intercept	32.6			
	Dizziness	− 6.4	− 10.4, − 2.4	0.3	0.002
	SOC-13 total score	0.7	0.5, 0.9		< 0.0001
	Heart disease	− 7.7	− 12.1, − 3.3		0.0008
	Vision impairment	− 5.7	− 9.8, − 1.7		0.006
	Joint/back problem	− 8.5	− 12.7, − 4.3		< 0.0001
Social functioning	Intercept	45			
	SOC-13 total score	0.6	0.4, 0.8	0.11	< 0.0001
	Joint/back problem	− 9.2	− 13.6, − 4.7		< 0.0001
Role emotional	Intercept	13.8			
	SOC-13 total score	1	0.6, 1.4	0.1	< 0.0001
	Diabetes	− 15.4	− 26.3, − 4.5		0.006
Mental health	Intercept	11.7			
	SOC-13 total score	1	0.8, 1.1	0.28	< 0.0001
	Joint/back problem	− 5.8	− 9.1, − 2.5		0.0007

Multivariable linear regression between different domains of Short form Health Survey questionnaire (SF-36) and sense of coherence (SOC-13), self-rated health (SRH), gender, conditions and diseases remaining after backward elimination process using exclusion criteria *p* > 0.01

Predictors included in the first step of the backward selection were female sex, dizziness, Sense of Coherence (SOC total score), diabetes, hypertension, hyper or hypothyroidism, lung disease (chronic bronchitis, asthma, chronic obstructive pulmonary disease), cardiac infarction/heart failure, prior stroke, vision or hearing impairment and joint/back problems

**Table 6:** Mean scores of the different domains in the Short form Health Survey questionnaire (SF-36) divided by sex

Mean (SD^a^) among women and men			
Domains	Total	Women	Men
	n = 467	n = 284	n = 185
Physical functioning	66.5 (25)	63.0 (25)	71.7 (23)**
Role physical	61.0 (42)	58.5 (43)	64.9 (41)
Bodily pain	65.1 (27)	61.4 (27)	70.9 (27)**
General health	63.5 (21)	62.9 (21)	64.4 (20)
Vitality	62.7 (23)	61.1 (24)	65.2 (22)
Social functioning	82.4 (24)	81.0 (24)	84.5 (23)
Role emotional	72.6 (39)	71.6 (40)	74.0 (37)
Mental health	79.0 (19)	76.7 (21)	82.6 (17)*

Mean scores of the different domains in the Short form Health Survey measuring health-related quality of life. The maximum score for SF-36 is 100 for every domain with higher scores indicating better health-related quality of life

^a^*SD* standard deviation. *p* values based on *t* test comparing men and woman, *p < 0.05; **p < 0.01

### SOC

There was no significant difference between dizzy and non-dizzy individuals in mean total scores of the SOC-13, nor when analyzing the subdomains, comprehensibility, manageability or meaningfulness, separately (Table 3). No sex differences regarding mean total score were observed (*p* = 0.94). In multiple linear regression, an association was observed between SOC and HRQL in all SF-36 domains except PF (Table 5).

### SRH and tiredness

A total of 73% of the dizzy participants reported feeling well, compared to 87% of the non-dizzy participants (Table 3). Half of the participants reporting dizziness (50%) felt generally tired compared to about one-third (32%) of the non-dizzy participants (*p* < 0.001) (Table 3). The odds ratio of feeling tired if reporting dizziness was 2.13 (CI 1.54–2.93, *p* < 0.0001) and 2.65 (CI 1.75–4.04, *p* < 0.0001) for not feeling well if reporting dizziness.

## Discussion

The aim of this study was to assess dizziness in relation to HRQL SOC, SRH and diseases among older adults living in an urban area in Sweden. We found that dizzy seniors were more tired and considered their HRQL and health to be poorer than non-dizzy seniors. There was a distinct difference in HRQL between dizzy and non-dizzy persons, where dizziness was associated with poorer HRQL particularly in the aspects of physical health.

In this study we found that HRQL was negatively influenced by having dizziness and dizziness together with joint/back problems and vision impairment were the conditions influencing HRQL the most in multivariate analyses. Having joint/back problems as well as vision impairment (cataracts) may themselves result in feeling unsteady and having balance problems [4, 36]. There was some correlation between SOC and HRQL but no significant difference in SOC between dizzy and non-dizzy participants.

Approximately half of the study population reported problems with dizziness or unsteadiness (54%), which is in line with previous reports from geriatric settings [37], but higher than previous research in Swedish settings [1]. Dizziness tends to be more common with increasing age as well as with higher morbidity and may well be a sign associated with morbidity, also in younger ages [5]. Having dizziness, non-vertigo type, has been associated with higher mortality risk, compared to dizziness due to peripheral causes [38]. The most common duration of dizziness was seconds and symptoms triggered by raising up or by positional changes, symptoms often seen in orthostatic hypotension or benign paroxysmal positional vertigo (BPPV). Dizziness and imbalance are also often associated with fear of falling as well as sometimes can be part of aging and frailty [4, 18]. Here, we found that participants with dizziness showed a lower HRQL than those without, including when adjusting for underlying comorbidities. Women in general tend to report poorer health and HRQL and seek medical care more often compared to men, and are prescribed more medications [39] 40, however, similar sex differences were not distinctly observed here, possibly due to the high age of the participants.

Knowledge of the impact of dizziness on HRQL among older adults is of great importance because the overall well-being and activity level may be hampered and this population may risk isolation due to fear of going out alone, as well as frustration and decreased self-esteem [17, 41]. Chronic dizziness may also lead to loss of confidence and restriction of physical and social activities to reduce risk of inconvenient symptoms.

The SF-36, although designed as a multidimensional tool to measure HRQL, targets mainly physical function and general health with less focus on mental health. The focus on function may be a reason why joint/back problems as well as vision impairment influenced HRQL so much in this study. The MH and RE domains are the two domains in SF-36 that evaluate the mental health aspects, of which the SF-36 MH domain showed the strongest correlation with SOC. This indicates that dizziness and impaired balance are associated with decreased HRQL in older people in aspects of physical function and general health rather than in mental or psychological parts. The enhanced level of ADL-dependency also indicates a decline in functional aspects among persons with dizziness.

In line with the findings of Mendel et al*.* investigating patients with vestibular disorders, having dizziness was not associated with lower mean total SOC score [29] in this population-based sample. The salutogenetic concept includes and combines many aspects of quality of life, including phycological and interpersonal and personal resources. As dizziness is so associated with comorbidity and decreased SRH and HRQL, an association between dizziness and total SOC-score could be expected, however not the case. Potentially because SOC focuses on less functional aspects of quality of life and more on personality traits. Antonovsky hypothesized that a high SOC may be a resource in creating positive health and may work as a buffer against life stressors, possibly seen as a protecting capacity at least if good health is preserved [35, 42]. In this cohort of 79-years-old mainly ordinary-home-dwelling individuals, we found an overall high level of SOC, higher than in other surveys investigating older seniors [43] and higher than in a middle-aged Swedish group investigated previously [44]. Also, SF-36 was high in relation to other reference age-related groups [45]. This high level of SOC, together with the high level of SRH indicates that the cohort is generally healthy and report being satisfied with their health situation.

Finally, one has to consider HRQL and health as complex constructs, which are influenced by many factors. Having dizziness showed negative associations with HRQL, tiredness and SRH, but not with SOC, possibly indicates that dizziness in old age mainly is associated with a decline in the self-perceived health status or the functional abilities of the person.

## Strengths and limitations

The strength of this study is the truly population-based sample selected through date of birth in the population registry. The examinations were performed by specially trained nurses or physicians. Due to the cross-sectional design of the study, the direction of the association cannot be established. No validated questionnaire for measuring dizziness were used Asking about both dizziness and unsteadiness in one question has some limitations as it results in asking two questions as one. Furthermore, the study is part of a multidisciplinary survey, which is why more participants completed the study questions about dizziness and general health than answered the validated SOC-13 (n = 452) and SF-36 questionnaires (n = 467).

## Conclusion

Dizziness was negatively associated with HRQL, also after adjusting for comorbidities. Identification and treatment of dizziness, when possible, are important because reduction of dizziness symptoms may potentially help to enhance overall well-being in this age group.

## Data Availability

Not applicable.
